# Gel-immersion-assisted laser ablation of a self-expandable metal stent using a novel 3-o’clock-channel cholangioscope

**DOI:** 10.1055/a-2791-4924

**Published:** 2026-02-24

**Authors:** Takeshi Ogura, Kimi Bessho, Junichi Nakamura, Nga Nguyen Trong, Hiroki Nishikawa

**Affiliations:** 1 38588 Pancreatobiliary Advanced Medical Center, Osaka Medical and Pharmaceutical University Hospital, Osaka, Japan; 2 38588 Endoscopy Center, Osaka Medical and Pharmaceutical University Hospital, Osaka, Japan; 3 13010 2nd Department of Internal Medicine, Osaka Medical and Pharmaceutical University, Osaka, Japan; 4 Department of Gastroenterology, Trong Nam Cancer Hospital, Ha Noi, Vietnam

After multiple self-expandable metal stent (SEMS) deployments, re-intervention for obstructed SEMSs has several issues 1 2 . First, dilation devices or stent insertion into the biliary tract through the mesh of a SEMS may be challenging. Second, although a cholangioscopic approach might be helpful, tumor ingrowth or debris may obstruct the cholangioscopic view. To overcome these issues, mesh ablation by laser and gel-immersion cholangioscopy may have benefits. However, ablation of the mesh of an occluded SEMS is challenging because the laser device is extracted from the 6-o’clock position. Limited space also hinders the operability of the cholangioscope. Recently, a cholangiopancreatoscope with the unique characteristic of providing the working channel exit at the 3-o’clock position (Briview, SeeGen Co., Ltd, Shanghai, China) has become available. Technical tips for gel-immersion-assisted laser ablation for SEMSs using this novel cholangioscope are presented.

**Fig. 1:**
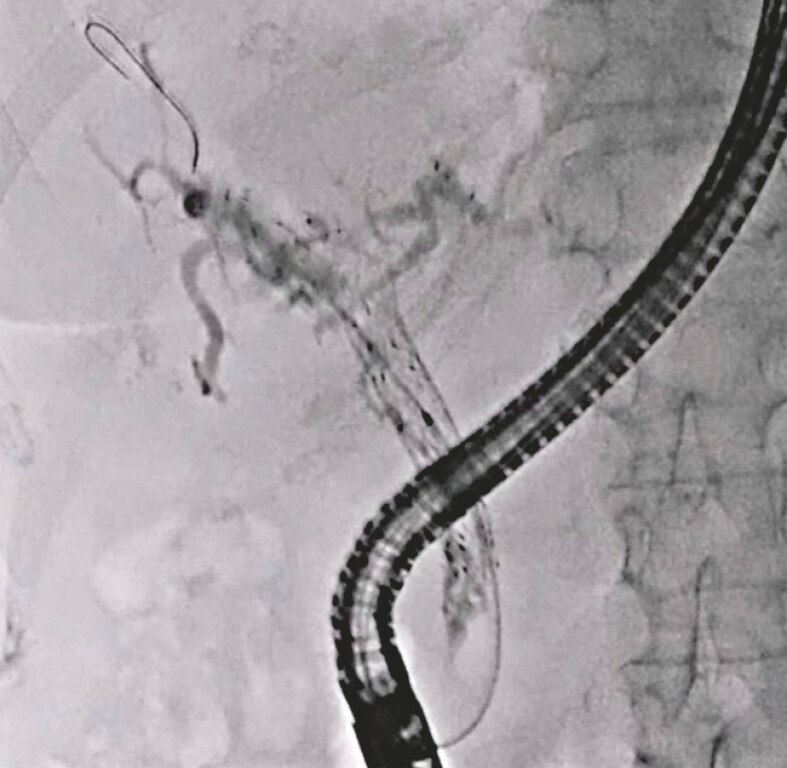
Obstruction of the self-expandable metal stent is observed.

**Fig. 2:**
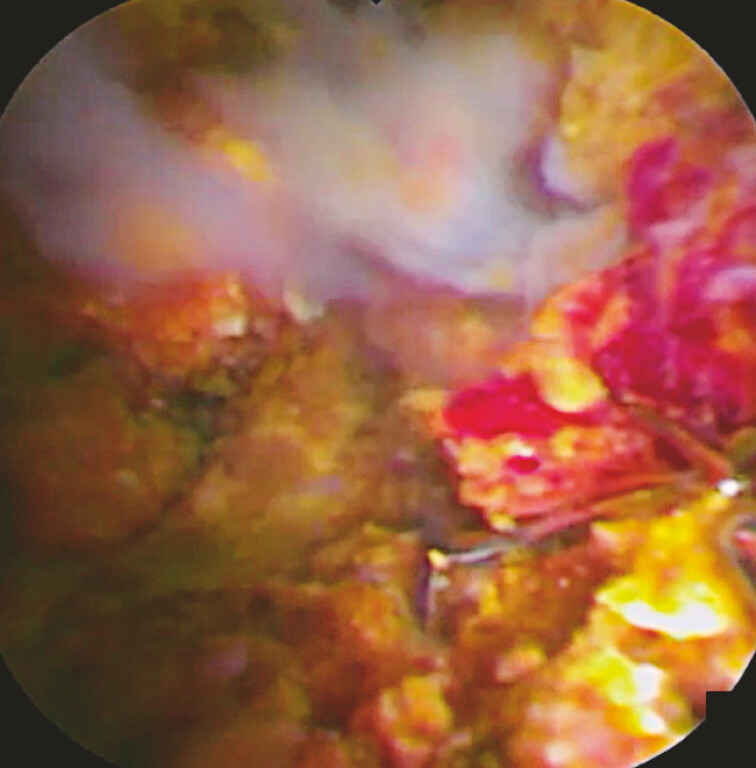
After gel injection, stones and the mesh of the self-expandable metal stent are clearly identified.

**Fig. 3:**
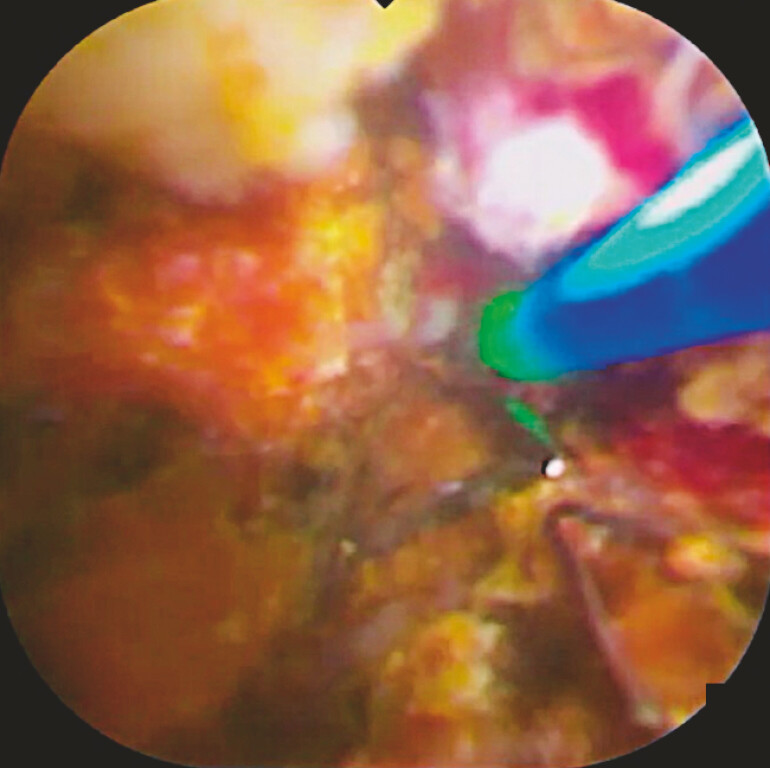
The laser probe is extracted from the 3-o’clock position.

**Fig. 4:**
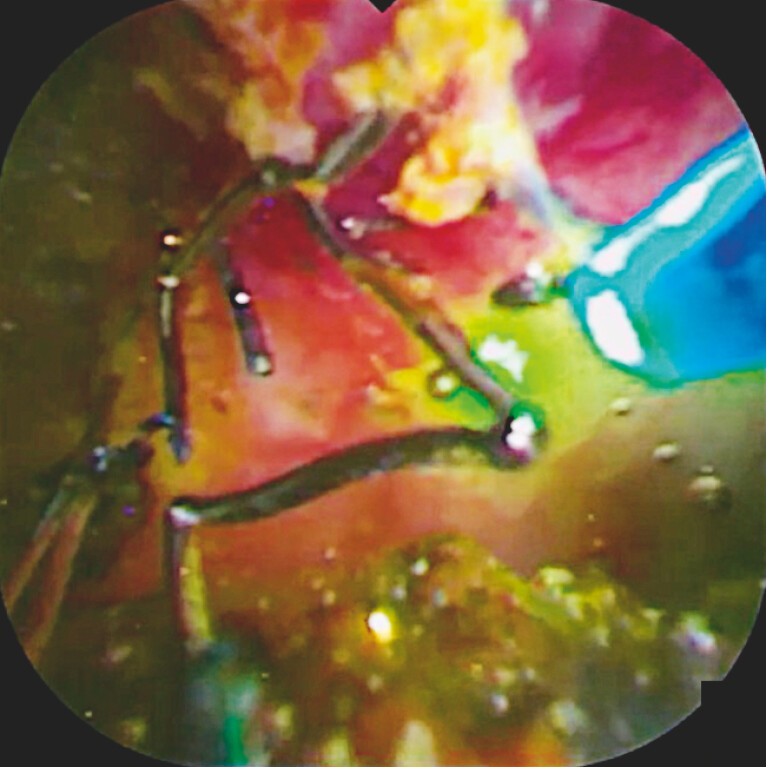
After laser ablation, the mesh of the self-expandable metal stent is broken, and the orifice of the anterior bile duct can be identified.

**Fig. 5:**
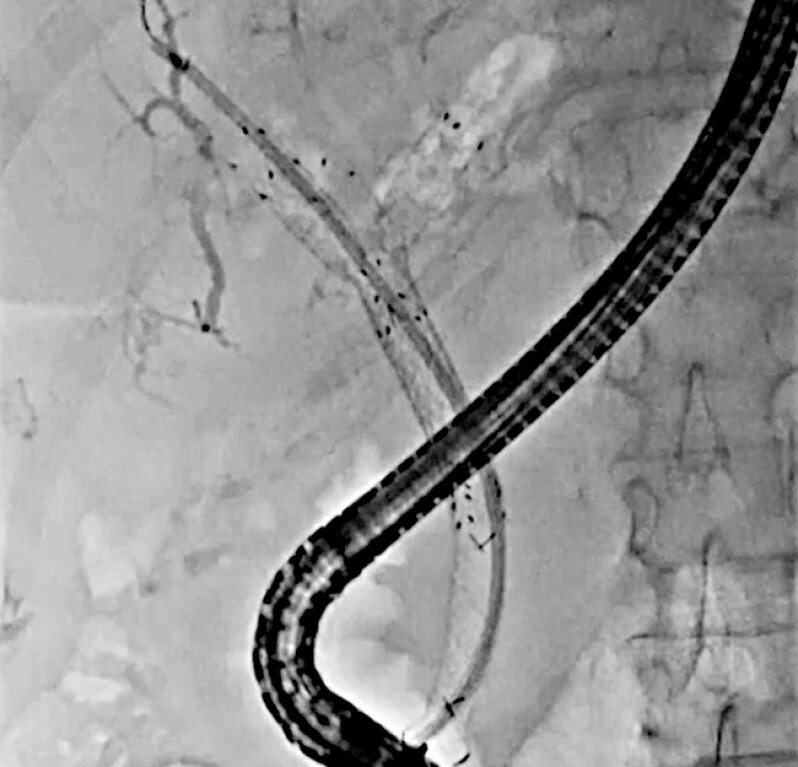
On re-intervention, a plastic stent can be deployed.

An 81-year-old woman underwent multiple uncovered SEMS deployments due to advanced cholangiocarcinoma. The serum bilirubin level was decreased from 11.1 mg/dL to 1.2 mg/dL. She underwent systematic chemotherapy; after 5 months, the serum bilirubin level was increased to 9.2 mg/dL, and based on computed tomographic imaging, anterior bile duct dilatation was observed. Therefore, re-intervention was attempted. First, an endoscopic retrograde cholangiopancreatography catheter was inserted into the biliary tract, and the contrast medium was injected. On cholangiography, anterior bile duct obstruction was observed ( Fig. 1 ). Because plastic stent insertion failed, peroral cholangioscopy was performed. The endoscopic view was extremely poor due to the presence of bile duct stones and debris. Following the injection of gel to improve the endoscopic view, the mesh of the SEMS was identified ( Fig. 2 ). Laser ablation of the mesh and laser lithotripsy for the bile duct stones were attempted ( Fig. 3 ). Since the laser probe was extracted from the 3-o’clock position in this scope, the procedures were performed easily. After breaking the mesh and fragmenting the stones, the orifice of the anterior bile duct was identified ( Fig. 4 ). Finally, a plastic stent was successfully deployed without any adverse events ( Fig. 5 , Video 1 ). After this procedure, the serum bilirubin level was decreased to 2.1 mg/dL, and the patient underwent systematic chemotherapy. After 4 months, although no adverse events including stent occlusion or cholangitis were observed, she was dead due to cholangiocarcinoma.

Gel-immersion-assisted laser ablation for a self-expandable metal stent using a novel 3-o’clock-channel cholangioscope is performed.Video 1

In conclusion, gel-immersion-assisted laser ablation for the SEMS using this novel cholangioscope may be useful.

Endoscopy_UCTN_Code_TTT_1AR_2AZ
